# MicroRNA-29b/c-3p Indicate Advanced Liver Fibrosis/Cirrhosis in Univentricular Heart Patients With and Without Fontan Palliation

**DOI:** 10.3389/fcvm.2020.619083

**Published:** 2021-01-08

**Authors:** Masood Abu-Halima, Eckart Meese, Mohamad Ali Saleh, Andreas Keller, Hashim Abdul-Khaliq, Tanja Raedle-Hurst

**Affiliations:** ^1^Institute of Human Genetics, Saarland University Medical Center, Homburg, Germany; ^2^Department of Pediatric Cardiology, Saarland University Medical Center, Homburg, Germany; ^3^Center for Clinical Bioinformatics, Saarland University, Saarbruecken, Germany

**Keywords:** microRNA, univentricular heart, MELD-Albumin score, ALBI score, collapsibility index

## Abstract

**Aim:** The present study aims to identify those microRNAs (miRNAs) in patients with univentricular heart (UVH) disease with and without Fontan palliation that may be associated with advanced liver fibrosis/cirrhosis.

**Materials and Methods:** SurePrint™ 8 × 60K Human v21 miRNA arrays were used to determine the miRNA abundance profiles in the blood of 48 UVH patients with and without Fontan palliation and 32 matched healthy controls. The abundance levels of selected miRNAs have been validated by quantitative reverse transcription-polymerase chain reaction (RT-qPCR).

**Results:** According to microarray analysis, 50 miRNAs were found to be significantly abundant in UVH patients of which miR-29b-3p and miR-29c-3p were significantly related to the model of end-stage liver disease (MELD)-Albumin and albumin-bilirubin (ALBI) score representing advanced liver fibrosis/cirrhosis. Relative expression levels of both miRNAs were significantly higher in patients with a higher collapsibility index representing venous hepatic congestion, a higher MELD-Albumin or ALBI score and incomplete or no Fontan palliation. In the logistic regression analysis, a MELD-Albumin score ≥ 11 or ALBI score > −2.6 were best predicted by total bilirubin (OR 6.630, *P* = 0.016), albumin (OR 0.424, *P* = 0.026), and miR-29c-3p (OR 33.060, *P* = 0.047). After adjustment to the status of Fontan palliation, however, no statistical significance of these parameters was found thus underlining the importance of palliation status on progression of liver fibrosis/ cirrhosis in UVH patients.

**Conclusions:** In UVH patients with and without Fontan palliation, miR-29b-3p and miR-29c-3p seem to be markers of advanced liver fibrosis/cirrhosis and thus may be used in the risk assessment of these patients.

## Introduction

Univentricular heart (UVH) disease is a rare and complex congenital heart disorder that is characterized by functionally single ventricle anatomy resulting in volume overload of the single ventricular chamber and systemic venous congestion. The only surgical option to improve hemodynamics in these patients is a palliative procedure called Fontan operation connecting both caval veins to the pulmonary artery and thus directing systemic venous blood return directly into the pulmonary circulation without the pulsatile function of a ventricular chamber. Depending on the pulmonary vascular resistance and diastolic function of the single ventricle, this passive venous blood flows into the pulmonary circulation results in chronic systemic venous congestion, particularly of the liver (1). In UVH patients with and without Fontan circulation, the development of liver fibrosis due to long-standing venous congestion of the liver is a well-known and challenging complication resulting finally in liver cirrhosis and in rare cases hepatocellular carcinoma (2–4). Thus, early detection of these complications in this cohort of patients is of diagnostic and prognostic importance. To date, liver biopsy still represents the gold standard in assessing the grade and stage of liver fibrosis. However, this technique is invasive and carries the potential risk of bleeding complications but also sampling and interpretation errors (5). Therefore, non-invasive diagnostic tools such as serological scoring or imaging-based methods should be used for screening and follow-up of congestive hepatopathy in these patients.

Using non-invasive methods, differentiation of reversible liver congestion from irreversible fibrosis and reliable prediction of liver fibrosis as assessed by biopsies remains challenging. Previous studies have shown that measurement of liver stiffness using transient elastography, shear wave elastography, or acoustic radiation force impulse imaging currently cannot differentiate reliably between congestion and fibrosis of the liver and thus may overestimate the extent of liver fibrosis in congestive hepatopathy (6, 7). Moreover, biochemical assessment of liver fibrosis using laboratory scores such as APRI, Forns, FIB4 or FibroSURE score is not feasible in these patients either because these scores do not correlate well with liver biopsy findings or are not related to the presence of sinusoidal or portal fibrosis on biopsy specimens that are typical for congestive hepatopathy (7, 8). However, the Model for End-Stage Liver Disease (MELD) excluding international normalized ratio (MELD-XI) score is a functional score indicating advanced liver disease that seems to be suitable in the risk assessment of patients with combined cardiac and hepatic dysfunction and even predicts clinical outcomes in congestive liver disease (6, 9, 10). Recently, a new MELD-Albumin score including albumin to replace INR in the conventional MELD score has been investigated in patients undergoing tricuspid annuloplasty for severe tricuspid regurgitation revealing to be a prognostic indicator as good as the MELD-XI score (11). Moreover, the ALBI score is applicable, robust, and superior to the conventional MELD score in the evaluation of the functional status and long-term prognosis of patients with liver cirrhosis (12, 13).

MicroRNAs (miRNAs) are known to be expressed in a cell type- or tissue-specific and stage-dependent manner in chronic liver disease (14–17). They also play a significant role in the regulation of liver fibrosis/cirrhosis representing the common end stage of most chronic liver diseases and being associated with tremendous morbidity and mortality (18, 19). Since miRNAs are known to modulate different steps in the pathophysiology of liver fibrosis, they might be used for early detection or progression of liver fibrosis (16). To our knowledge, no data are available on the specific miRNAs that are involved in the onset or progression of liver fibrosis in UVH patients. In this study, we aimed to identify miRNAs that indicate significant liver fibrosis in UVH patients using prognostic scores of advanced liver fibrosis/cirrhosis, specifically the new MELD-Albumin and ALBI score, and to assess the most important determinants of advanced liver fibrosis/cirrhosis in this cohort of patients.

## Materials and Methods

### Patients

Between 02/05/2015 and 18/06/2018, 48 consecutive UVH patients who were regularly seen in our outpatient clinic were enrolled in the present study. 42/48 (87.5%) patients had a complete Fontan palliation and 6/48 (12.5%) patients an incomplete or no Fontan palliation. Regarding the morphology of the dominant systemic ventricle, 32/48 (66.7%) patients had a morphological dominant left, and 16/32 (50%) patients a morphological dominant right systemic ventricle. In the left systemic ventricle UVH group, 12 patients presented with tricuspid atresia, 12 patients with double inlet left ventricle, and eight patients with pulmonary atresia with or without ventricular septal defect. In the right systemic ventricle UVH group, eight patients had hypoplastic left heart syndrome or mitral atresia, and eight patients double outlet right ventricle with pulmonary stenosis. The mean age was 22.8 ± 10.1 years (range 11– 46 years) including 17 females and 31 males. At enrollment, a structured protocol including a 12-lead surface electrocardiogram, a physical examination, measurement of blood pressure and transcutaneous oxygen saturation at rest, two-dimensional echocardiography as well as a venous blood draw for routine laboratory parameters and blood sampling were performed. Additionally, ultrasonographic parameters of liver congestion such as the diameter of the inferior caval vein (IVC) at deep inspiration and expiration were measured to calculate the collapsibility index (20). Clinical characteristics of the patient cohort and UVH-specific data have already been presented in a previous study (21). Patients' specific liver parameters are illustrated in Table 1. Thirty-two healthy volunteers served as controls and were matched to UVH patients according to age and gender. In all volunteers, a physical examination, two-dimensional echocardiography to verify the absence of any heart abnormality, ultrasonography of the inferior caval vein to rule out liver congestion, and venous blood sampling were performed on the same day. The study complies with the Declaration of Helsinki, was approved by the local ethics committee and all participants or their guardians gave written and informed consent before enrollment.

**Table 1:** Specific liver parameters of UVH patients according to the status of palliation.

**Variables**	**All patients (*n* = 48)**	**Complete Fontan palliation (*n* = 42)**	**Incomplete or no Fontan palliation (*n* = 6)**	*P-*value^*^
Age at enrolment (years)	22.8 ± 10.1	20.5 ± 8.5	38.2 ± 4.3	<0.001
Male gender (%)	31/48 (64.6%)	29/42 (69.0%)	2/6 (33.3%)	ns
NYHA class	1.5 ± 0.7	1.4 ± 0.6	2.7 ± 0.5	<0.001
Diameter of IVC at deep inspiration (mm)	14.9 ± 4.3	14.3 ± 3.9	18.8 ± 5.6	0.038
Collapsibility index of IVC	0.27 ± 0.09	0.28 ± 0.09	0.19 ± 0.06	0.018
Collapsibility index of IVC ≤ 0.15	6/48 (12.5%)	3/42 (7.1%)	3/6 (50%)	0.020
Total bilirubin (mg/dl)	0.75 (0.60–0.98)	0.70 (0.60–0.90)	1.05 (0.80–2.38)	0.021
Albumin (g/l)	48.0 (44.0–49.0)	48.0 (44.0–49.0)	43.5 (37.8–49.0)	ns
γGT (U/l)	67.0 (40.5–96.0)	67.5 (45.8–102.0)	36.5 (22.8–70.8)	0.025
Platelet count (per mm^3^)	193.5 (152.0–230.0)	200.0 (166.3–238.3)	147.5 (123.5–187.0)	0.034
Creatinine (mg/dl)	0.79 (0.66–0.94)	0.78 (0.64–0.91)	0.99 (0.78–1.38)	ns
GFR (ml/min)	105.9 (85.3–122.5)	107.8 (96.8–125.3)	82.4 (60.8–92.1)	0.007
MELD-XI score	9.44 (9.44–10.51)	9.44 (9.44–9.44)	11.24 (9.44–15.95)	0.014
MELD-Albumin score	6.43 (6.43–7.40)	6.43 (6.43–7.17)	8.42 (7.11–12.89)	0.003
ALBI score	−3.25 (−3.41–−2.97)	−3.27 (−3.42–−3.03)	−2.79 (−3.33–−2.36)	0.057
MELD-Albumin score ≥ 11 or ALBI score > −2.6 (%)	5/48 (10.4%)	2/42 (4.8%)	3/6 (50%)	0.010

*UVH, univentricular heart; GFR, glomerular filtration rate; MELD, model of end-stage liver disease; MELD-XI, model of end-stage liver disease without international normalized ratio; ALBI; albumin-bilirubin; ns, not significant*.

*Mean ± standard deviation or median (interquartile interval) are used*.

* *Complete compared to incomplete/no Fontan palliation subgroup*.

### Sample Preparation and RNA Isolation

In all patients and controls, blood samples for miRNA detection were collected in PAXgene™ blood tubes (Becton–Dickinson, Heidelberg, Germany) shortly after echocardiographic evaluation. All PAXgene™ blood tubes were stored at room temperature for at least 24 h to ensure complete lysis of the blood cells, then stored at −20°C for several days and finally transferred to −80°C for long-term storage until RNA isolation. Total RNA including miRNAs was isolated from blood samples using PAXgene™ Blood miRNA Kit on the QIAcube™ robot (Qiagen, Hilden, Germany) following the manufacturer's recommendations and included DNase I treatment (Qiagen). To confirm the absence of genomic DNA contamination, a conventional PCR with exon spanning primers for Glyceraldehyde 3-Phosphate Dehydrogenase (*GAPDH*) as previously described (22). The concentration of isolated total RNA was measured using the NanoDrop ND-2000 spectrophotometer (Thermo Fisher Scientific, Massachusetts, United States). RNA purity was estimated by examining the OD 260/280 and the OD 260/230 ratios. The qualities of total RNA were assessed using the Agilent Bioanalyser 2100 Eukaryote Total RNA Nano Series II (Agilent Technologies, California, United States).

### Analysis of miRNAs by Microarray

The miRNA abundance profile from the 48 patients with UVH and 32 age- and gender-matched healthy controls was obtained from our previously generated and uploaded raw data to the NCBI GEO database (Accession ID: GSE136547) using SurePrint™ 8X60K Human v21 miRNA platforms (Agilent Technologies) (21). These platforms contain probes for the detection of 2,549 human miRNAs. An input amount of 100 ng of isolated RNA including miRNAs was labeled and subsequently hybridized to the miRNA microarray chip and the procedure was completed as previously described (23).

### Analysis of miRNAs by RT-qPCR

Real-time quantitative PCR (RT-qPCR) validation analysis was performed using the StepOnePlus™ Real-Time PCR System (Applied Biosystems, Foster City, CA, United States) and the *mi*Script PCR System (Qiagen) as previously described (24). For the validation step, we used the same samples that were used for the microarray analysis (48 UVH patients and 32 healthy controls) (21). Fifteen miRNAs (miR-101-3p, miR-144-5p, miR-18a-5p, miR-15a-5p, miR-17-3p, miR-96-5p, miR-18b-5p, miR-140-5p, miR-148a-3p, miR-29b-3p, miR-29c-3p, miR-210-3p, miR-125a-5p, miR-150-5p, and miR-324-3p) were chosen for RT-qPCR validation based on the significant abundance changes on the microarray analysis. Of these miRNAs, miR-29b-3p and miR-29c-3p were significantly correlated to the prognostic scores of advanced liver fibrosis/cirrhosis (MELD-Albumin and ALBI score). For miRNA abundance level detection, an amount of 250 ng of the total RNA was converted into complementary DNA (cDNA). The resulting cDNA was then diluted to have 0.5 ng/μL input material for miRNA detection. All RT-qPCR experiments were carried out using the Liquid Handling Robot QIAgility™ (Qiagen) before performing RT-qPCR. All primer assays used in the current study were provided by Qiagen. Moreover, miRNA reverse transcription control (miRTC) (Qiagen) was performed to assess the performance of the reverse transcription reaction. The melting curve analysis was used to control the specificity of RT-qPCR products. The specificity of amplicons was further confirmed by agarose gel electrophoresis.

### Data Analysis

Clinical data of the patients were collected from medical records. Echocardiography and ultrasonography were performed using a Vivid™ E9 Ultrasound System (GE Healthcare, Horten, Norway). The echocardiographic loops and ultrasonographic images were stored digitally and analyzed on an Echopac server (Echopac Version 6, GE Healthcare). The collapsibility index of the IVC was calculated according to the formula: the difference of maximum and minimum diameter of the IVC divided by the maximum diameter of the IVC (20). The formulas used for calculating MELD and modified MELD scores have been described previously (11). ALBI score was calculated according to the equation: ALBI score = (log_10_ bilirubin × 0.66) + (albumin × −0.085) and has been previously published (25). Echocardiographic and ultrasonographic data sets were assessed by investigators blinded to the laboratory results. Investigators of miRNA signatures were blinded to the clinical, echocardiographic, ultrasonographic, and laboratory data of the patients.

### Statistical Analysis

DataAssist™ Software v3.0 (Applied Biosystems) was used to calculate the relative abundance changes of miRNAs by the equation 2^−Δ*Ct*^ with RNAU6B serving as an endogenous control as previously used and validated for this type of sample (21, 23, 24, 26–31). Adjustment for multiple testing was performed by controlling the false discovery rate (FDR) according to the approach of Benjamini and Hochberg (32). Clinical data were analyzed using standard statistical software (SPSS version 25; SPSS Inc., Chicago, Illinois). Continuous variables are expressed as mean ± standard deviation or median (interquartile interval) as appropriate. Differences between unpaired groups were analyzed using a Mann-Whitney-U test for continuous variables and a chi-square test (or Fisher exact test, if numbers were small) for nominal variables. Correlations were evaluated using Spearman's regression coefficient. Univariate and multivariate analysis was performed using binary logistic regression to identify the most significant factors of advanced liver fibrosis/cirrhosis. Variables entered into the multivariate model were those that gave statistically significant results in the univariate analysis and didn't show any multicollinearity. A two-tailed *P* < 0.05 was considered statistically significant.

## Results

### Evaluation of Conventional Liver Parameters

Conventional ultrasonographic and laboratory liver parameters according to the status of Fontan palliation are presented in Table 1. As compared to patients with complete Fontan palliation, patients with incomplete or no Fontan palliation had a higher NYHA class, a lower glomerular filtration rate, and a lower collapsibility index of the IVC reflecting the presence of more severe global heart failure and liver congestion. Moreover, laboratory parameters of advanced liver fibrosis/cirrhosis such as total bilirubin or platelet count were also different between both subgroups. The calculated prognostic liver scores MELD-XI, MELD-Albumin, and ALBI scores were significantly higher in patients without Fontan palliation indicating more advanced stages of liver fibrosis/cirrhosis.

### Identification of Abundant miRNAs

**Figure 1:**
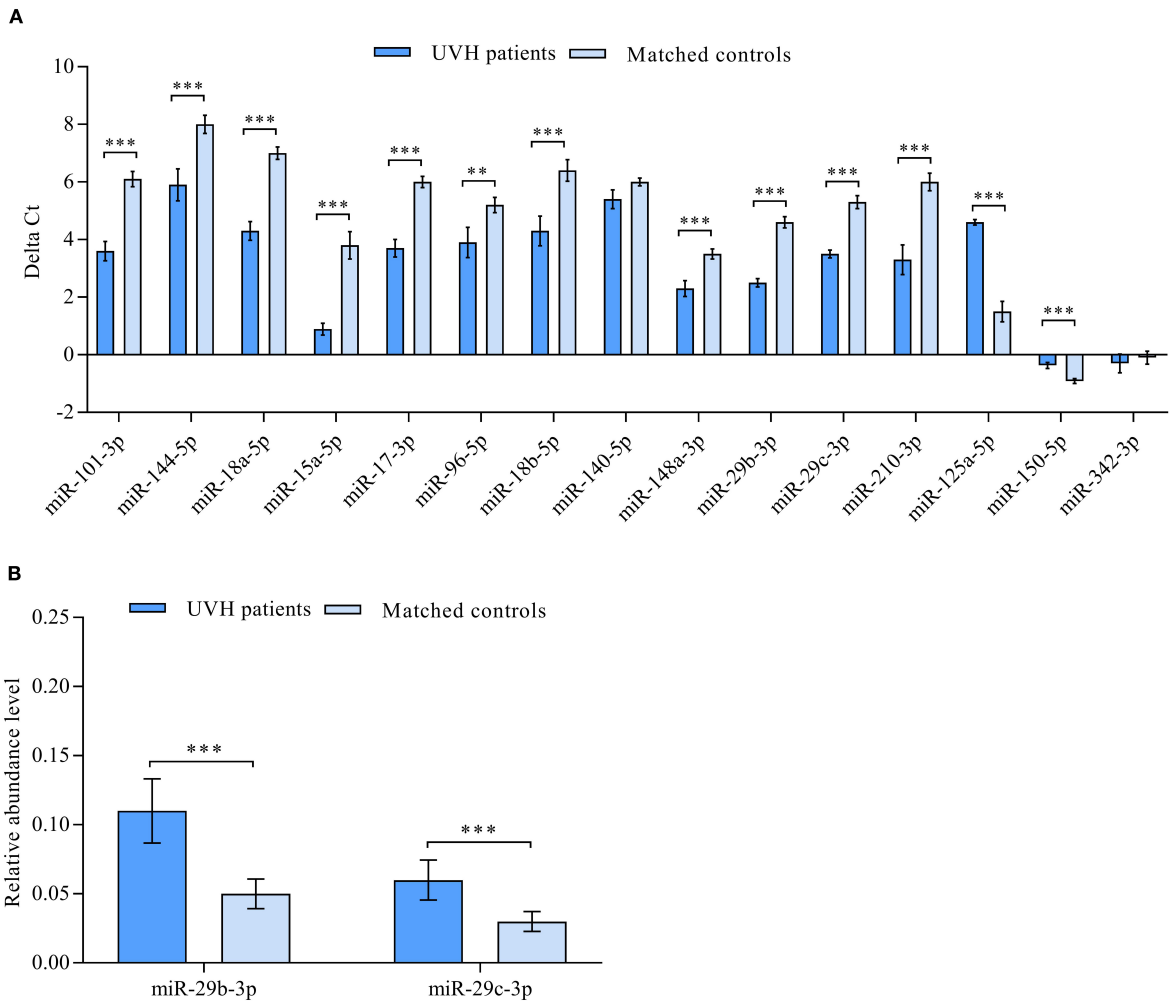
**(A)** Validation of differentially abundant miRNAs in the blood of patients with univentricular heart (UVH) (*n* = 48) compared to age and gender-matched healthy controls (*n* = 32) as determined by RT-qPCR (*P* < 0.05). Mean ΔCt values of patients and healthy controls *(lower* Δ*Ct values indicating higher abundance levels)*. **(B)** Validation of clinically correlated differentially abundant miR-29b-3p and miR-29c-3p in the blood of UVH patients (*n* = 48) compared to age and gender-matched healthy controls (*n* = 32) as determined by RT-qPCR (*P* < 0.05). Mean relative abundance level (2^−ΔCt^) of patients and healthy controls are shown with RNAU6B as an endogenous control for normalization. Un-paired-two-tailed *t*-tests and mean ± standard deviation (STDV) were used to evaluate differences in abundance levels. **P ≤ 0.01; ***P ≤ 0.001.

According to a miRNA microarray, a total of 50 miRNAs were found to be differentially abundant when comparing the samples from patients with UVH to healthy controls (21). RT-qPCR confirmed the direction of abundance changes and the significance of different abundances for 13 miRNAs. Specifically, out of 15 miRNAs used for the RT-qPCR validation, 11 miRNAs showed statistically significant higher abundance levels and two miRNAs showed statistically significant lower abundance levels in UVH patients compared with those in healthy controls (*P* < 0.05, FDR adjusted) (Figure 1A). There was no significant difference in the abundance level of miR-140-5p and miR-342-3p (Figure 1A). However, correlation analyses of significantly abundant miRNAs to prognostic scores of advanced liver fibrosis/cirrhosis (MELD-Albumin and ALBI score) identified miR-29b-3p and miR-29c-3p to be best related to these scores. Thus, these two miRNAs were further validated by RT-qPCR indicating significantly higher abundance levels in UVH patients compared to healthy controls (Figure 1B).

### Association of miRNAs With Measures of Liver Congestion and Prognostic Liver Fibrosis Scores

**Figure 2:**
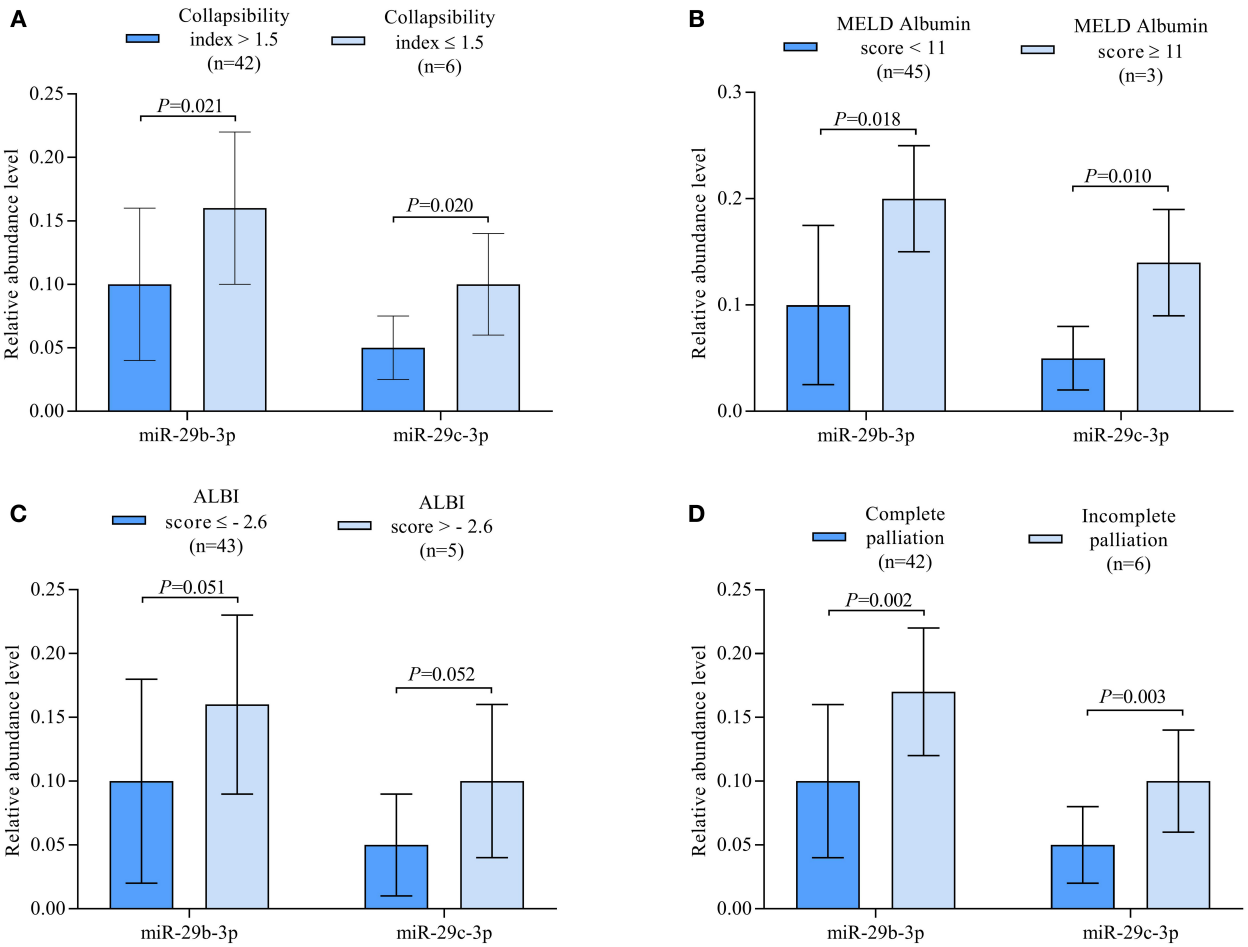
**(A)** Boxplots illustrating relative abundance levels of miR-29b-3p and miR-29c-3p in UVH patients with (*n* = 6) and without (*n* = 42) severe liver congestion as indicated by a collapsibility index of the IVC ≤ 0.15. **(B)** Boxplots illustrating normalized expression levels of miR-29b-3p and miR-29c-3p in UVH patients with a MELD-Albumin score < 11 (*n* = 45) and ≥ 11 (*n* = 3) indicating advanced liver fibrosis/cirrhosis. **(C)** Boxplots illustrating normalized expression levels of miR-29b-3p and miR-29c-3p in UVH patients with an ALBI score ≤ −2.6 (*n* = 43) and > −2.6 (*n* = 5) indicating a higher degree of liver cirrhosis. **(D)** Boxplots illustrating normalized expression levels of miR-29b-3p and miR-29c-3p in UVH patients with complete (*n* = 42) and incomplete (*n* = 6) Fontan palliation. UVH, univentricular heart; IVC, inferior caval vein.

Correlation analysis revealed miR-29c-3p to be significantly related to the extent of liver congestion represented by a collapsibility index of the IVC ≤ 0.15, laboratory measures of severe liver dysfunction such as total bilirubin and platelet count as well as prognostic measures of advanced liver fibrosis/cirrhosis as indicated by the different MELD scores and ALBI score. In contrast, miR-29b-3p was only significantly related to albumin and ALBI score (Table 2). Relative abundance levels of miR-29b-3p and miR-29c-3p were significantly higher in patients with severe liver congestion as indicated by a collapsibility index of the IVC ≤ 0.15 (Figure 2A). Moreover, higher relative abundance levels of miR-29b-3p and miR-29c-3p were observed in patients with a higher MELD-Albumin score ≥ 11 and ALBI score > −2.6 both indicating advanced liver fibrosis/cirrhosis (Figures 2B,C) as well as in patients with incomplete or no Fontan palliation (Figure 2D).

**Table 2:** Correlation of miRNAs validated by RT-qPCR with liver-specific parameters and prognostic liver scores (*n* = 48).

**Variables**	**miR-29b-3p**		**miR-29c-3p**	
	***r***	***P*-value**	***r***	***P-*value**
Collapsibility index of IVC	−0.325	0.024	−0.359	0.012
Collapsibility index of IVC ≤ 0.15	0.336	0.020	0.340	0.018
Total bilirubin (mg/dl)	0.310	0.032	0.387	0.007
Albumin (g/l)	−0.366	0.010	−0.288	0.047
γGT (U/l)	–	ns	–	ns
Platelet count (per mm^3^)	–	ns	−0.330	0.022
MELD-XI score	0.295	0.042	0.361	0.012
MELD-Albumin score	0.285	0.050	0.330	0.022
ALBI score	0.447	0.001	0.384	0.007
Death from any cause	0.283	0.051	0.276	0.058

*IVC, inferior caval vein; MELD, model of end-stage liver disease; MELD-XI, model of end-stage liver disease without international normalized ratio; ALBI, albumin bilirubin; ns, not significant*.

### Indicators of Advanced Liver Fibrosis/Cirrhosis

Logistic regression analysis was used to identify the most significant factors of advanced liver fibrosis/cirrhosis as indicated by a MELD-Albumin score ≥ 11 or an ALBI score > −2.6 in all patients (Table 3). Here, univariate analysis identified total bilirubin, albumin and miR-29c-3p to be the most important factors of advanced liver fibrosis/cirrhosis. After adjustment for palliation status, however, no statistical significance could be seen thus indicating the importance of palliation status for advanced liver fibrosis/cirrhosis in UVH patients.

**Table 3:** Determinants of advanced liver fibrosis/cirrhosis according to combined MELD-Albumin score ≥ 11 or ALBI score > −2.6.

	**Univariate analysis**			Multivariate analysis^*^		
**Variables**	**OR**	**95% CI**	***P-*value**	**OR**	**95% CI**	***P-*value**
Total bilirubin (mg/dl)	6.630	1.423–30.884	0.016	6.560	0.978–43.993	0.053
miR-29b-3p	20.944	0.671–653.432	0.083	6.279	0.072–549.233	0.421
Albumin (g/l)	0.424	0.199–0.901	0.026	0.280	0.051–1.532	0.142
Platelets (per mm^3^)	0.991	0.975–1.008	0.291	1.000	0.981–1.020	0.990
miR-29c-3p	33.060	1.044–1046.655	0.047	26.803	0.343–2092.997	0.139

*OR, odds ratio; CI, confidence interval*.

* *Adjusted for status of Fontan palliation*.

## Discussion

In UVH patients with and without Fontan palliation, the development of liver fibrosis/cirrhosis due to chronic liver congestion is a typical late complication (2, 3). These non-cardiac complications may limit potential heart transplantation candidacy and overall survival in this cohort of patients with advancing age. Thus, in addition to cardiorespiratory parameters, monitoring of liver status during the long-term follow-up of these patients is crucial. However, currently used methods to assess the grade or stage of liver fibrosis in these patients show significant limitations (6, 8). Recently, specific miRNAs are dysregulated in different liver diseases (18, 19) but are also involved in hepatic fibrosis progression (16) and thus might be used as valuable biomarkers. Therefore, our study aimed to identify those miRNAs in UVH patients with and without Fontan circulation that are associated with advanced liver fibrosis/cirrhosis and to assess the most significant determinants of advanced liver fibrosis/cirrhosis in this cohort of patients.

Using conventional ultrasonographic and laboratory parameters such as collapsibility index and different scores of liver fibrosis/cirrhosis, we were able to demonstrate abnormal liver findings in our cohort of patients that were more severe in UVH patients with incomplete or no Fontan palliation as compared to patients with complete Fontan palliation (Table 1). Moreover, UVH patients without Fontan palliation were significantly older and presented with a higher NYHA class reflecting more severe systolic and diastolic dysfunction of the UVH in these patients. In contrast to published data indicating Fontan physiology as the leading cause of liver morbidities, we were able to show that UVH patients without Fontan palliation may also suffer from the same liver complications in older age.

In this study, miR-29b-3p and miR-29c-3p were found to be best associated with the prognostic liver scores MELD-Albumin and ALBI score in our patients using microarray analysis. After validation by RT-qPCR, miR-29b-3p turned out to be significantly related to ALBI score whereas miR-29c-3p was best related to MELD-Albumin, MELD-XI, and ALBI score (Table 2). It is of note that these liver scores represent advanced stages of liver fibrosis or even liver cirrhosis and also have prognostic value in patients with chronic liver and heart disease. In contrast to previous studies evaluating these miRNAs in other clinical settings, both miRNAs were upregulated in our patient cohort with or without Fontan palliation as compared to healthy controls (Figure 1B). However, it needs to be taken into consideration that the pathomechanism of liver fibrosis/cirrhosis after inflammatory or toxic liver damage may be different from congestive liver disease that was present in our studied patients. Moreover, higher abundance levels of both miRNAs were found in patients with a higher MELD-Albumin or ALBI score indicating advanced stages of liver fibrosis/cirrhosis (Figures 2B,C). Furthermore, abundance levels of both miRNAs were significantly elevated in UVH patients with incomplete or no Fontan palliation reflecting the more longstanding venous congestion of the liver, more advanced stages of liver fibrosis/cirrhosis, and a poorer prognosis in these patients as compared to patients with complete Fontan palliation (Figure 2D). Overall, both miRNAs were able to differentiate between lower and higher liver fibrosis/cirrhosis scores indicating the progression of liver pathology and worsening of prognosis.

Our results are in accordance with previous studies identifying the miR-29 member family as an indicator of advanced liver fibrosis/cirrhosis. In a previous liver biopsy study in patients with hepatitis B-associated liver fibrosis, a panel of 4 miRNAs was identified to be involved in the progression of liver fibrosis, namely miR-122, miR-146a-5p, miR-29c-3p, and miR-223 (33). In that study, miR-122 has been shown to differentiate between various fibrosis stages (F0-F4) and thus can be used as a precise fibrosis staging biomarker, especially for the early detection of liver fibrosis. In contrast, miR-29c-3p has been shown to be only significantly abundant in advanced liver fibrosis/cirrhosis in that study and is in agreement with previous studies identifying the miR-29 family as a marker of advanced liver fibrosis/cirrhosis (34, 35).

An increasing number of studies have demonstrated the down-regulation of miR-29 in human fibrotic disorders of multiple organs by transforming growth factor ß/*SMAD3* signaling (34–36). Moreover, miR-29 has been identified as a strong tumor suppressor rather than an oncogenic factor in most cancer studies (37) and many of these studies indicate that high miR-29 expression leads to increased survival (38, 39). However, there are also some studies reporting that higher levels of miR-29 in plasma are associated with lower survival rates (40, 41) and that up-regulation of miR-29c may be associated with higher relapse risk in patients with acute myeloid leukemia (42). In our study, the correlation of both miRNAs to all-cause mortality was statistically borderline (Table 2) what can be explained by the fact that mortality was mainly cardiac-related in this cohort of patients (21). Nevertheless, liver fibrosis and cirrhosis are important complications increasing with age and limiting survival in UVH patients with or without Fontan palliation. In our study, the elevated abundance levels of miR-29b-3p and miR-29c-3p in UVH patients with incomplete or no Fontan palliation may indicate the potential worse overall prognosis and limited survival in these patients (Figure 2D).

### Study Limitations

This is the first study that aims to characterize signatures of miRNAs in UVH patients with and without Fontan palliation using a large panel of miRNAs for initial screening in order to identify those that may indicate advanced liver fibrosis/cirrhosis and thus might have clinical as well as prognostic impact in this cohort of patients. Since UVH disease is a rare congenital cardiac disorder, the sample size of our patient cohort is rather small. Moreover, non-invasive methods that reliably diagnose different stages of liver fibrosis in this cohort of patients are limited (6, 8). Thus, prognostic scores of advanced liver fibrosis/cirrhosis were used for miRNA screening in the present study. The cross-sectional rather than longitudinal design of the study may limit the predictive value of the measured miRNAs. Therefore, the measured miRNAs may have limited value in detecting earlier stages of liver fibrosis in such patients. Hence, a larger cohort of UVH patients should be evaluated and liver biopsies performed to assess the diagnostic value of miRNAs in the early and late stages of liver fibrosis in these patients.

## Conclusions

In UVH patients with and without Fontan palliation, miR-29b-3p and miR-29c-3p are associated with measures of advanced liver fibrosis/cirrhosis and thus may be used as potential biomarkers in the risk assessment of these patients.

## Data Availability Statement

The datasets generated for this study can be found in online repositories. The names of the repository/repositories and accession number(s) can be found in the article/supplementary material.

## Ethics Statement

The studies involving human participants were reviewed and approved by Institutional Review Board approval/Ethikvotum Ärztekammer des Saarlandes: Ethical vote No. 73/09. Written informed consent to participate in this study was provided by the participants' legal guardian/next of kin. The animal study was reviewed and approved by Ethical vote No. 73/09. Written informed consent was obtained from the owners for the participation of their animals in this study.

## Author's Note

This manuscript has been released as a pre-print at Research Square (43).

## Author Contributions

MA-H performed experimental work, particularly the miRNA isolation, array experiment, RT-qPCR validation, and helped in manuscript writing. EM designed the study, coordinated the molecular biology experiment, and edited the manuscript. MS helped in the experimental work and analyses. AK performed bioinformatics analysis. HA-K designed the study and diagnosed patients. TR-H designed the study, recruited and examined controls, diagnosed patients, collected blood samples, and helped in writing. All authors read and approved the final manuscript.

## Conflict of Interest

The authors declare that the research was conducted in the absence of any commercial or financial relationships that could be construed as a potential conflict of interest.
